# 173. Staphylococcus Aureus Bacteremia at a Community Hospital in the Southeastern United States June 2021-June 2022

**DOI:** 10.1093/ofid/ofad500.246

**Published:** 2023-11-27

**Authors:** Gretchen S Arnoczy, Heather Gibson, Kimberly Starr

**Affiliations:** FirstHealth of the Carolinas, Pinehurst, North Carolina; FirstHealth of the Carolinas, Pinehurst, North Carolina; Moore Regional Hospital, Pinehurst, North Carolina

## Abstract

**Background:**

*Staphylococcus aureus b*acteremia is a common condition with high risk for mortality. Recent COVID-19 infection may influence the incidence and outcome of *S. aureus* bacteremia.

**Methods:**

We performed a retrospective chart review of individuals with *Staphylococcus aureus* bacteremia from June 2021-June 2022 in a rural community health care system in the Southeastern United States. Primary outcome was mortality—variables included methicillin resistance, persistent bacteremia, ID consult, history of injection drug use, cardiac imaging, and COVID-19 infection within the previous 90 days.

**Results:**

172 individuals experienced 188 episodes of *Staphylococcus aureus* bacteremia. 79 were MRSA, 99 were MSSA. 57 individuals were deceased within 6 months of the positive blood culture (33% mortality). 31 individuals had known injection drug use complicating their illness, endocarditis was confirmed in 18 (58%) of these individuals compared to 33 (19%) of the entire population. 116 individuals (67%) had an ID consult. This study occurred during the delta and omicron COVID-19 surges in the region – 18 episodes of *S. aureus* bacteremia occurred within 90 days of a positive COVID-19 test, and 10 (56%) of those individuals were deceased within 6 months of the positive blood culture.

**Conclusion:**

*S. aureus* bacteremia carries a high risk for mortality. Endocarditis was confirmed more frequently in individuals with known injection drug use. Six month mortality in *S. aureus* bacteremia was higher when bacteremia occurred in the 90 days post COVID-19 infection.

**Disclosures:**

**All Authors**: No reported disclosures

